# Hydroxylamine production by *Alcaligenes faecalis* challenges the paradigm of heterotrophic nitrification

**DOI:** 10.1126/sciadv.adl3587

**Published:** 2024-06-07

**Authors:** Wouter B. Lenferink, Lars R. Bakken, Mike S. M. Jetten, Maartje A. H. J. van Kessel, Sebastian Lücker

**Affiliations:** ^1^ Department of Microbiology, Radboud Institute for Biological and Environmental Sciences, Radboud University, Heyendaalseweg 135, 6525 AJ, Nijmegen, Netherlands.; ^2^ Faculty of Chemistry, Biotechnology and Food Science, Norwegian University of Life Sciences, 1432 Ås, Norway.

## Abstract

Heterotrophic nitrifiers continue to be a hiatus in our understanding of the nitrogen cycle. Despite their discovery over 50 years ago, the physiology and environmental role of this enigmatic group remain elusive. The current theory is that heterotrophic nitrifiers are capable of converting ammonia to hydroxylamine, nitrite, nitric oxide, nitrous oxide, and dinitrogen gas via the subsequent actions of nitrification and denitrification. In addition, it was recently suggested that dinitrogen gas may be formed directly from ammonium. Here, we combine complementary high-resolution gas profiles, ^15^N isotope labeling studies, and transcriptomics data to show that hydroxylamine is the major product of nitrification in *Alcaligenes faecalis*. We demonstrated that denitrification and direct ammonium oxidation to dinitrogen gas did not occur under the conditions tested. Our results indicate that *A. faecalis* is capable of hydroxylamine production from an organic intermediate. These results fundamentally change our understanding of heterotrophic nitrification and have important implications for its biotechnological application.

## Teaser

Heterotrophic nitrification produces hydroxylamine from an organic intermediate, without subsequent biological conversion to N_2_.

## INTRODUCTION

Nitrogen is a key element for life on Earth. It is present in many molecules such as proteins and nucleic acids. The most abundant form of nitrogen is dinitrogen gas (N_2_), which however is not available to most organisms. Instead, they take up more reactive forms of nitrogen, mainly ammonium (NH_4_^+^). While reactive nitrogen is often limiting in natural ecosystems, its anthropogenic input continues to increase (*1*). This can have detrimental effects on ecosystems, as an excess of reactive nitrogen causes eutrophication and the release of greenhouse gases such as nitrous oxide (N_2_O) (*2*). Therefore, there is a need for continued efforts to understand the microbial transformations in the biogeochemical nitrogen cycle.

NH_4_^+^ is the primary source of nitrogen for most organisms. In its deprotonated form, ammonia (NH_3_) is also used as an energy source by nitrifying bacteria and archaea (*3*). Nitrification is the stepwise oxidation of NH_3_ via hydroxylamine (NH_2_OH) to nitrite (NO_2_^−^) and further to nitrate (NO_3_^−^). In soils, NH_4_^+^ is relatively well retained by the negatively charged soil particles. However, the negatively charged NO_2_^−^ and NO_3_^−^ ions are more susceptible to runoff and leaching, and nitrification thus impairs fertilizer efficiency and increases eutrophication of receiving water bodies. NO_3_^−^ can be lost from the environment through denitrification, the stepwise reduction of NO_3_^−^ to NO_2_^−^, nitric oxide (NO), N_2_O, and lastly N_2_ (*4*). Denitrifiers use the intermediates of this pathway as alternative electron acceptors instead of oxygen (O_2_). Thus, denitrification is a common anaerobic respiration strategy for microorganisms in oxygen-limited environments. The existing body of research on nitrification suggests that this is primarily an autotrophic process (*3*, *5*, *6*) performed by ammonia-oxidizing bacteria (AOB) and archaea (AOA), and nitrite-oxidizing bacteria. In addition, complete ammonia oxidizers (comammox) accomplish both steps within a single organism (*7*, *8*). It is well-recognized that substantial amounts of N_2_O can be produced during nitrification (*5*, *9*, *10*). In part, this is due to a process called nitrifier denitrification, which occurs under low O_2_ availability. However, a full canonical denitrification pathway is absent from known nitrifiers, and large parts of the N_2_O production are probably due to detoxification of NH_2_OH by cytochrome P460 (*11*).

More controversial is the physiology of heterotrophic nitrifiers. Some soil bacteria such as *Pseudomonas aeruginosa* (*12*) and *Arthrobacter siderocapsulatus* (*13*, *14*) have been found to produce NO_2_^−^ from NH_2_OH and aldoximes (*15*, *16*). Others, such as *Arthrobacter globiformis* (*17*), *Paracoccus pantotrophus* (*18*), *Alcaligenes faecalis* (*19*), and *Pseudomonas putida* (*20*) were found to produce NH_2_OH, NO_2_^−^, and NO_3_^−^ from NH_4_^+^. The intense dispute surrounding heterotrophic nitrification results from the lack of understanding of the underlying biology. In AOB and AOA, ammonia (NH_3_) is oxidized via the sequential actions of ammonia monooxygenase (AMO) and hydroxylamine dehydrogenase (HAO). Energy is conserved from the electrons released during the oxidation of NH_2_OH to NO_2_^−^. In contrast, this could not be verified for NH_4_^+^ or NH_2_OH oxidation in heterotrophic nitrifiers (*21*, *22*). Nonetheless, several publications report the isolation of AMO- and HAO-like proteins from *P. pantotrophus* (*23*–*25*), *Pseudomonas* PB16 (*26*), *A. faecalis* (*27*), and *Acinetobacter* Y16 (*28*). AbrB-family transcriptional regulators have been suggested as putative AMO proteins due to a proposed structural similarity in *P. putida* (*20*). However, a recent study in *A. faecalis* showed that *abrB* knockout mutants did not lose the ability to oxidize NH_3_ (*29*). Still, it is commonly presumed that heterotrophic nitrification occurs through a similar mechanism as in canonical nitrifiers (*30*).

Strikingly, apart from *A. globiformis*, most described heterotrophic nitrifiers were also reported to be capable of denitrification under oxic conditions. Robertson and Kuenen (*31*) proposed a role for heterotrophic nitrification to generate electron acceptors for aerobic denitrification. The rationale for this theory is that denitrification under oxygen-limited conditions could stimulate faster turnover of reduced nicotinamide adenine dinucleotide (NADH). The measured effect of this strategy was an increase in growth rate, albeit at the cost of growth yield. Some heterotrophic nitrifiers were found to be capable of simultaneous heterotrophic nitrification and aerobic denitrification (HNAD) (*32*, *33*). This results in the removal of NH_4_^+^ through denitrification, without additional biomass yield. This trait is regarded as particularly interesting for biological nitrogen removal in high nitrogen-containing wastewater treatment systems (*34*). For example, *A. faecalis* was reported capable of efficiently removing nitrogen from high-strength NH_4_^+^ wastewater by HNAD (*35*, *36*).

*A. faecalis* was first described over a century ago (*37*, *38*) and is commonly found in soil, freshwater, and wastewater environments. On multiple occasions, *A. faecalis* was found to nitrify NH_4_^+^, thereby producing NH_2_OH, NO_2_^−^, NO_3_^−^, NO, N_2_O, and N_2_ (*19*, *33*, *39*). Contrary to expectations, NH_2_OH accumulated in these cultures, and it has been claimed that the oxidation of NH_2_OH is therefore of little significance (*40*). The formation of NO_2_^−^ is argued to be largely pH dependent (*40*, *41*) and may be linked to oxime formation. Free NH_2_OH can react with aldehyde groups of small organic molecules, for instance forming pyruvic oxime from pyruvate. It was found that *A. faecalis* uses an enzyme called pyruvic oxime dioxygenase (POD) to oxidize pyruvic oxime, yielding pyruvate and NO_2_^−^ (*42*, *43*). Furthermore, Tsujino and coworkers (*42*) showed that POD homologs are found in several proteobacteria capable of heterotrophic nitrification, which could therefore be an explanation for the observed production of NO_2_^−^ by heterotrophic nitrifiers.

The exact mechanism by which *A. faecalis* mediates the conversion of inorganic nitrogen remains a subject of debate, and the nature of NO, N_2_O, and N_2_ formation still is uncertain. *A. faecalis* was shown to be capable of denitrification under aerobic conditions (*33*). However, this was not found to occur simultaneously with heterotrophic nitrification (*19*, *44*). Instead, Otte and coworkers (*44*) suggested that NO_2_^−^ was not denitrified, but N_2_O was a direct byproduct of NH_2_OH oxidation and was subsequently reduced to N_2_. Recent research suggests that *Alcaligenes* species are capable of direct NH_4_^+^ oxidation to N_2_, with NH_2_OH as intermediate (*45*, *46*). This direct oxidation of NH_4_^+^ and NH_2_OH is thought to be catalyzed by a process termed direct nitrogen formation (DNF), encoded by the *dnfABCD* gene cluster. The *dnfA* gene encodes a diiron oxygenase and was reported to mediate the oxygen-dependent conversion of NH_4_^+^ or NH_2_OH. In agreement with this hypothesis, Xu and coworkers (*29*) reported that *dnfAB* knockout mutants of *A. faecalis* were unable to produce NH_2_OH and, therefore, N_2_. However, purified DnfA was demonstrated to bind NH_2_OH but not NH_4_^+^ (*47*), and recent research proposed a function in the hydroxylation of the amide group of glutamine to form glutamic acid hydroxamate in addition to NH_2_OH oxidation to N_2_ (*48*). Hence, the role of the Dnf system in NH_4_^+^ and NH_2_OH oxidation remains to be investigated further.

In this study, we set out to characterize the nitrogen turnover reactions occurring in *A. faecalis*. We used a complementary combination of advanced cultivation methods and gas analysis in combination with ^15^N isotope labeling, genomics, and transcriptomics. Thereby, we deliver a comprehensive overview of the inorganic nitrogen metabolism of *A. faecalis*. Furthermore, we provide strong indications that the reported DNF by *A. faecalis* is likely due to abiotic processes.

## RESULTS

### Genome reconstruction and annotation

To enable transcriptome studies, the genome of *A. faecalis* American Type Culture Collection (ATCC) 8750 was sequenced. Hybrid assembly of long- and short-read sequencing data yielded a 4,072,866–base pair (bp) circular genome of *A. faecalis* ATCC 8750 with 124× coverage, a G+C content of 56.6 mole percent (mol %), and a total of 3718 coding sequences (CDSs). The genome contained three complete ribosomal RNA operons and 58 transfer RNAs, one to five for each of the 20 amino acids. Our assembly compared well to that of other *A. faecalis* genomes (16 complete genomes, as of February 2023), which, on average, were 4.16 Mb in size, had a G+C content of 56.6 mol %, and contained 3724 CDSs. The presence of plasmids was not investigated, which has been reported for some strains (*49*).

NH_4_^+^ uptake and assimilation are encoded by an NH_4_^+^ transporter gene (*amtB*), two genes encoding the glutamate dehydrogenase (*gdhA*), and the glutamine synthetase/glutamate 2-oxoglutarate aminotransferase pathway (*glnA* and *gltBD*). These pathways are regulated by a set of nitrogen-regulatory P-II proteins (GlnB>, GlnD, GlnE, and GlnK). In addition, global control of the nitrogen and carbon metabolisms of the cell is mediated by the phosphotransferase system (PTS) and PTS_Ntr_ systems (RpoN, PtsN, PtsO/NPr, ManX, PtsI, and PtsH, but not PtsP).

Genes homologous (>90% amino acid identity) to the *dnf* cluster in *Alcaligenes ammonioxydans* (*45*) are present in *A. faecalis*. This cluster comprises *dnfABCD* and the upstream *dnfT1T2RT3*. Also, POD and two homologs (PODh1 and PODh2) with 44.5 and 48.2% amino acid identity to POD, respectively, were identified. An AbrB family transcriptional regulator is encoded, which had been proposed to be involved in NH_4_^+^ oxidation in *P. putida* (*20*). However, as mentioned above, the function of the AbrB protein as AMO was recently disproven (*29*).

In addition, the genome of *A. faecalis* contained all genes necessary for denitrification from NO_2_^−^ to N_2_, but not for NO_3_^−^ reduction to NO_2_^−^. Genes for NO_2_^−^ and quinol-dependent NO reduction are colocalized on the genome (*nirK* and *norBR*, respectively), followed by a Crp/Fnr family transcriptional regulator resembling NnrR. N_2_O reduction is encoded by *nosRZDFYL*. We did not find evidence of a NirS-type nitrite reductase, in contrast to another strain of *A. faecalis* (*50*).

*A. faecalis* has multiple genes that deal with nitrosative stress. Next to nitric oxide reductase (*norZ*), the genome also contains a cytochrome *bd*–type oxidase and a flavohaemoprotein, encoded by *cydAB* and *hmp*, respectively. Cytochrome *bd*–type oxidases have been implicated with nitrosative stress resistance due to their high NO dissociation rate (*51*). Furthermore, *katA* and *katE* encode catalases that may be involved in NO_2_^−^ detoxification to NO_3_^−^ (*52*, *53*). Glutathione production and salvage are encoded by *gshA*, *gshB*, and *ggt* and are involved in numerous stress responses.

### Growth and gas production under nitrifying conditions

*A. faecalis* produced N_2_O and N_2_ in batch incubations under all tested conditions (fig. S1), concurrent with CO_2_ production (fig. S2). In addition, NO production was observed under carbon-to-nitrogen (C/N) ratios of 8 and 4. Incubations that contained NH_2_OH evolved N_2_O and N_2_ before the onset of CO_2_ production, indicating the abiotic decay of NH_2_OH (fig. S1). The final optical densities at 600 (OD_600_) were 0.203 ± 0.02, 0.463 ± 0.02, and 0.557 ± 0.04 for C/N 20, 8, and 4, respectively.

### ^15^N stable isotope labeling

**Fig. 1.**
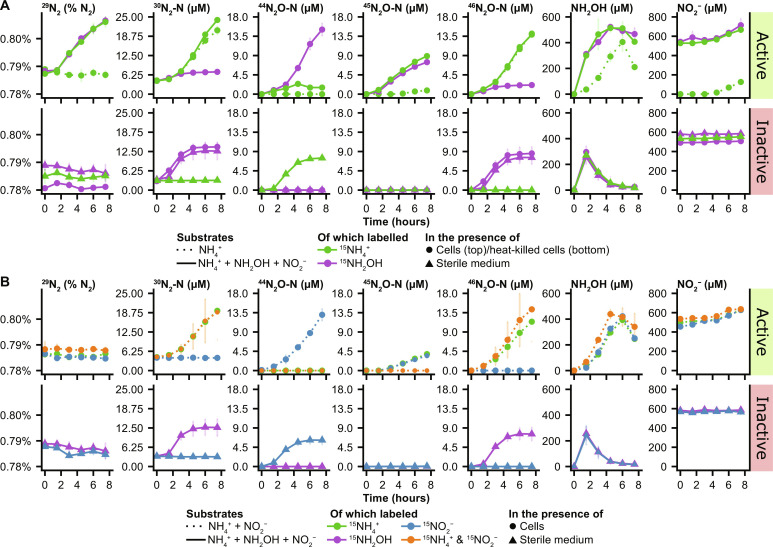
Inorganic nitrogen metabolism of *Alcaligenes faecalis* and abiotic reactions of NH_2_OH. Results are shown for *A. faecalis* grown in the presence of stable isotope-labeled nitrogen compounds. NH_2_OH was added to the respective incubations at *t* = 1. (**A**) Incubations performed in the presence of NH_4_^+^ (dotted lines) or NH_4_^+^, NH_2_OH, and NO_2_^−^ (solid lines), amended with ^15^N-labeled ^15^NH_4_^+^ (green) or ^15^NH_2_OH (purple). (**B**) Incubations with NH_4_^+^ and NO_2_^−^ (top; dotted lines) and additionally with NH_2_OH (bottom; solid lines), amended with ^15^N-labeled ^15^NH_4_^+^ (green), ^15^NH_2_OH (purple), ^15^NO_2_^−^ (blue), or ^15^NH_4_^+^ and ^15^NO_2_^−^ (orange). Data points and error bars represent the mean and SDs, respectively, of three biological replicates.

Nitrification and growth of *A. faecalis* were observed under all conditions where NH_4_^+^ was added and were not inhibited by the addition of NO_2_^−^ or NH_2_OH (Fig. 1 and fig. S3). Again, the production of NH_2_OH, NO_2_^−^, N_2_O, and N_2_ coincided with the production of CO_2_. Incubations supplied with NH_4_Cl as the sole source of nitrogen consumed in total 47 ± 2 μmol NH_4_^+^ and produced up to 6.5 ± 1.2 μmol NH_2_OH, 1.9 ± 0.2 μmol NO_2_^−^, 1.6 ± 0.1 μmol N_2_O-N, and 1.7 ± 0.1 μmol N_2_-N.

NH_2_OH was produced from NH_4_^+^ and reacted in a biomass-independent manner to yield N_2_ and N_2_O, but not NO_2_^−^ (Fig. 1A). In sterile incubations, NH_2_OH reacted with itself to form ^44^N_2_O and presumably ^28^N_2_ (not measured) when ^14^NH_2_OH was supplied or ^46^N_2_O and ^30^N_2_ when ^15^NH_2_OH was supplied. Cells fed with ^15^NH_2_OH in the presence of ^14^NH_4_^+^ additionally produced ^44^N_2_O, ^45^N_2_O, and ^29^N_2_, resulting from the biological conversion of ^14^NH_4_^+^ to ^14^NH_2_OH (Fig. 1A). No reaction was observed between NH_2_OH and NO_2_^−^ under sterile conditions (Fig. 1A).

The production of ^46^N_2_O and ^30^N_2_ was dependent on the addition of ^15^NH_2_OH or ^15^NH_4_^+^ (Fig. 1B). Denitrification did not occur, as indicated by the lack of conversion of ^15^NO_2_^−^ to ^46^N_2_O and ^30^N_2_. However, active cells fed with ^15^NO_2_^−^ and ^14^NH_2_OH produced ^45^N_2_O, which was not observed under sterile conditions. This suggests that NO_2_^−^ was reduced to NO, which subsequently reacted with NH_2_OH to form N_2_O.

The abiotic reaction of NO with NH_2_OH was verified under sterile oxic conditions, where ^14^NO declined rapidly, leading to the production of ^45^N_2_O in the presence of ^15^NH_2_OH (fig. S4). ^46^N_2_O and ^30^N_2_ were formed independently of NO disappearance, and ^30^N_2_ production was higher in mineral medium (MM) than in ddH_2_O. Furthermore, ^46^N_2_O production occurred only in MM, showing that this reaction depends on constituents of the medium, most likely metals.

### Chemostat culture

**Fig. 2.**
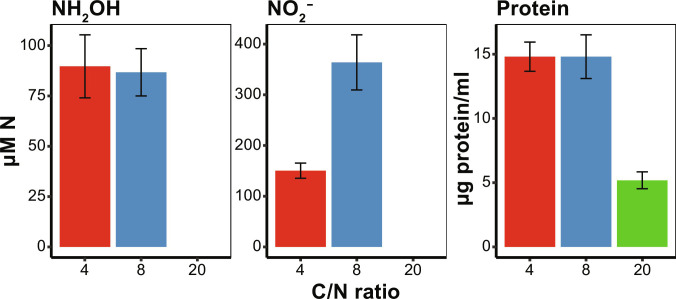
Inorganic nitrogen production and growth of *A. faecalis* in chemostat culture at different C/N ratios. Shown are steady-state concentrations of NH_2_OH (**Left**) and NO_2_^−^ production (**Middle**) and protein yield (**Right**) at C/N ratios of 4 (red), 8 (blue), and 20 (green). Data points and error bars represent the mean and SDs, respectively, of three technical replicates.

Cells cultured in continuous culture produced 87 ± 12 and 90 ± 16 μM NH_2_OH, and 364 ± 54 and 150 ± 15 μM NO_2_^−^ at C/N 8 and 4, respectively (Fig. 2). NO_3_^−^ concentrations remained under the detection limit of the assay (2.5 μM) but might have been masked by the high NO_2_^−^ concentrations. No production of NH_2_OH and NO_2_^−^ was observed at C/N 20. The protein yields at C/N 4 and 8 were comparable (14.8 ± 1.1 and 14.8 ± 1.7 μg protein ml^−1^, respectively), while at C/N 20, the culture reached a lower protein content (5.2 ± 0.7 μg protein ml^−1^), indicating that the cells were experiencing nitrogen limitation.

### Differential gene expression

Gene expression was compared between steady-state reactor cultures at incremental NH_4_^+^ concentrations at three different C/N ratios. In total, 332 unique genes were differentially expressed [log_2_ fold change (LFC) ≥ 1.5, false discovery rate (FDR) ≤ 0.05; table S1]. A total of 261 genes were differentially expressed between C/N 8 and 20, 291 genes between C/N 4 and 20, and 9 genes between C/N 4 and 8.

**Fig. 3.**
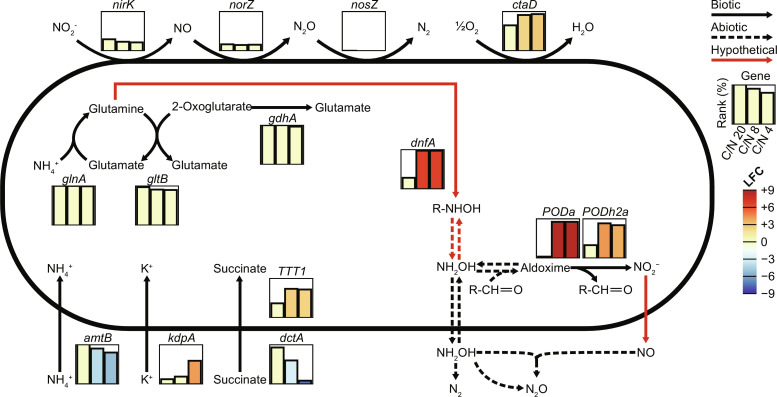
Differential gene expression analysis and pathways of (in)organic nitrogen metabolism in *A. faecalis*. The rank (% of all CDSs) of selected genes is shown for three C/N ratios. Colored bars indicate that a gene is differentially regulated compared to the C/N 20 condition. Red lines are hypothetical reactions discussed in this work. R-NHOH may be represented by glutamic acid monohydroxamate. LFC, log_2_ fold change.

In the nitrifying conditions C/N 8 and 4, most genes of the DNF, POD, and PODh2 clusters were highly up-regulated (Fig. 3), as well as several transaminases, hydroxymethyltransferases, and acetyl-CoA transferases involved in amino acid biosynthesis. In addition, the *caa3*-type cytochrome *c* oxidase was up-regulated, but not the *bd*-type quinol oxidase.

Markedly, numerous up-regulated genes encoded parts of transport complexes. This included 10 clusters encoding putative ABC transporters for amino acids and dipeptides, 9 individual clusters of high-affinity tripartite ATP-independent periplasmic family-related transporters, 1 high-affinity tricarboxylate transporter of the TTT family, and 1 CstA-like transporter of the putative carbon starvation family. Specific for the C/N 4 condition compared to both C/N 8 and 20 was the up-regulation of the K^+^-translocating ATPase gene cluster *kdpFACBDE*, and the down-regulation of an ABC transporter putatively involved in amino acid transport.

Genes for NH_4_^+^ uptake (*amtB* and its regulator *glnK*), the glutathione salvage protein Ggt, and the putative low-affinity C_4_-dicarboxylate transporter DctA were down-regulated in both C/N 8 and 4 compared to C/N 20. This further strengthened that conditions C/N 8 and 4 were carbon-limited, while the C/N 20 condition was limited in nitrogen.

Last, a cluster of seven poorly characterized genes (AFN_v2_0075-AFN_v2_0081) was up-regulated at C/N 8 and 4. This cluster encodes a putative oxidoreductase, acyl-CoA ligase, and thiolase that share similarities to proteins involved in carnithine and benzoate metabolism. In addition, one of these gene products contains a rubredoxin-like zinc ribbon domain (DUF35_N).

## DISCUSSION

Heterotrophic nitrification is generally reported to occur under a wide range of C/N ratios. For example, one study found that *A. faecalis* nitrifies in batch cultures growing on citrate or acetate at C/N ratios 28, 14, and 7, which was confirmed at C/N 20, 10, and 5 (*54*, *55*). In agreement with these results, we found that *A. faecalis* nitrifies when grown in batch on succinate and NH_4_^+^ at C/N 20, 8, and 4 and when grown in a chemostat culture at C/N 8 and 4. Strikingly, nitrification was not observed when *A. faecalis* was grown in chemostat cultures at C/N 20. Under this condition, the supplied NH_4_^+^ was not measurable in the supernatant and was most likely limiting the growth of *A. faecalis*, as also reflected by the lower protein yield. The C/N 20 condition also showed the highest transcription of the AmtB-type high-affinity NH_4_^+^ transporter. In contrast, conditions C/N 8 and 4 showed down-regulation of AmtB and up-regulation of aminotransferases and other proteins involved in amino acid biosynthesis. Moreover, the observed up-regulation of K^+^ uptake is in line with a regulatory response to nitrogen sufficiency (*56*).

In contrast, batch incubations with NH_4_^+^ only reach an N-limited state after all NH_4_^+^ is consumed. This is reflected by the decrease in N_2_O and N_2_ production (fig. S2) and a net decrease in NH_2_OH concentration (fig. S3) after respiration stops. At C/N 8 and 4, N_2_O and N_2_ production were coupled to respiration. At C/N 20, the rate of N_2_O and N_2_ production decreased before respiration stopped, which is arguably caused by a limitation in NH_4_^+^. Therefore, it can be assumed that *A. faecalis* produces NH_2_OH in a state of nitrogen excess. However, this is not the case for NO_2_^−^ production, which continues probably due to the remaining NH_2_OH.

The inorganic nitrogen metabolism of *A. faecalis* has been heavily debated. Classically, *A. faecalis* is thought to perform HNAD (*33*). However, Otte and coworkers (*44*) reported that denitrification could not explain N_2_O and N_2_ formation when *A. faecalis* was nitrifying NH_2_OH. In agreement with these findings, we could not identify denitrification as a source of N_2_O and N_2_. Our ^15^N-labeling experiments unequivocally showed that NO_2_^−^ was not converted to N_2_O and N_2_ by *A. faecalis* under nitrifying conditions. These results are further corroborated by knockout studies performed in *A. faecalis*, where cells with inactivated *nirK* or *nosZ* were still capable of N_2_ production during nitrification (*29*).

One unanticipated finding in our study was the production of ^45^N_2_O by *A. faecalis* in incubations with NH_4_^+^ and NO_2_^−^ when one of these compounds was ^15^N-labeled (Fig. 1B). While this accumulation of ^45^N_2_O further confirmed the absence of NosZ activity, which would have resulted in ^29^N_2_ formation, the origin of the ^45^N_2_O in these incubations remains unclear. We argue that its formation could be explained by a chemical reaction between NH_2_OH and NO (Fig. 3) and demonstrate that NH_2_OH and NO react rapidly to form N_2_O under the experimental conditions, even if it should be noted that the concentrations used in this abiotic assay far exceed their expected fluxes encountered in nature (fig. S4). Furthermore, NO production by *A. faecalis* during nitrification has been reported on multiple occasions (*19*, *57*) and was observed in our batch incubations (fig. S1). How *A. faecalis* forms NO under these conditions is uncertain. The *nirK* gene was among the lowest 25% expressed genes during nitrification, and its expression did not change significantly upon exposure to NO_2_^−^. Still, low-level constitutive activity of NirK could explain NO production. Alternatively, NO formation from NO_2_^−^ has also been observed by “nondedicated” NO_2_^−^ reductases, such as certain molybdoenzymes (*58*).

The accumulation of NH_2_OH is indicative of an imbalance between its production and consumption. Our results seem to agree with previous work of Castignetti and coworkers (*21*), who found that *A. faecalis* did not appreciably metabolize NH_2_OH to NO_2_^−^. Instead, they found that *A. faecalis* could produce NO_2_^−^ from pyruvic oxime, which was thought to be an intermediate in nitrification at the time. Pyruvic oxime forms in a spontaneous reaction between NH_2_OH and pyruvic acid. However, it should be noted that the occurrence of pyruvic oxime in *A. faecalis* cultures has not been studied.

Nevertheless, the increase in POD transcription in nitrifying *A. faecalis* (Fig. 3) substantiates that pyruvic oxime is present and could be the source of NO_2_^−^ production. Xu and coworkers (*29*) recently reported that knockout mutants of *A. faecalis* POD were still able to produce NO_2_^−^, which likely can be explained by the presence of several homologs of POD. Consistently, Tsujino and coworkers (*59*) showed that one of these homologs (designated PODh2 in our study) was also capable of pyruvic oxime oxidation, albeit at 5.5% of the rate of POD. This homolog was also significantly up-regulated during NH_2_OH production in our study.

It is commonly suggested that heterotrophic nitrification happens through the concerted action of AMO- and HAO-like proteins, analogous to autotrophic ammonia oxidation (*30*, *60*). Furthermore, AMO- and HAO-like proteins have been isolated from heterotrophic nitrifiers in a few instances (*20*, *23*–*26*). However, except for the refuted AMO-like AbrB-family protein in *P. putida*, no sequences are available. In this investigation, we also were unable to find evidence for AMO- and HAO-like proteins in the genome of *A. faecalis* based on sequence homology. Instead, it was recently proposed that the genus *Alcaligenes* is capable of direct NH_4_^+^ oxidation via NH_2_OH to N_2_ (*46*). Consistent with these findings, we found that nitrogen from ^15^NH_2_OH ends up in ^46^N_2_O and ^30^N_2_. Furthermore, when ^15^NH_2_OH was present in addition to ^14^NH_4_^+^, ^45^N_2_O and ^29^N_2_ were formed, supporting the notion that NH_4_^+^ is first converted to NH_2_OH before N_2_O and N_2_ are produced. However, we were unable to demonstrate that N_2_O and N_2_ formation were biotic processes. In our experiments, the production of NH_2_OH from NH_4_^+^ unequivocally required the activity of *A. faecalis*. However, despite the up-regulation of the *dnf* genes, NH_2_OH accumulated in the medium, and the oxidation rate of NH_2_OH was similar between active cells and control incubations (Fig. 1 and fig. S3), strongly suggesting the notion that N_2_O and N_2_ formation were abiotic. The disappearance of NH_2_OH appeared to be a first-order reaction, being proportional to the NH_2_OH concentration. These kinetics have been observed before but were ascribed to enzymatic activity (*44*).

Our findings renew the debate on the nature of NH_2_OH oxidation by *A. faecalis*, which may be explained by the reactivity of NH_2_OH. Here, we find abiotic transformation of NH_2_OH to N_2_O and N_2_ and of NH_2_OH with NO to N_2_O, which is in agreement with previous reports (*61*, *62*). These abiotic conversions are especially stimulated by trace metals and O_2_ (*62*, *63*). Consistent with this, we found that the abiotic conversion of NH_2_OH to N_2_ proceeded faster in MM than in ddH_2_O, and N_2_O formation was completely absent in ddH_2_O. This strongly indicates that the production of N_2_O from NH_2_OH relies on the presence of metals such as Cu and Fe, while N_2_ production is only stimulated by this.

Heterotrophic nitrification has been speculated to function in maintaining the metabolic redox balance. The “bottleneck” theory proposed that HNAD serves as an electron sink for faster NADH turnover during rapid growth (*32*). Wu and colleagues (*45*) reason a similar function for DnfA, which is hypothesized to use NADH as an electron donor via DnfB (*47*). The idea of nitrification as a redox balancing mechanism is tempting but would rely on the saturation of the NADH pool. Yet, heterotrophic nitrification seems to be favored in conditions where carbon appears limiting in comparison to the nitrogen pool. Moreover, while heterotrophic nitrification is tightly linked to respiration, it ultimately depends on a surplus of nitrogen, as shown in our chemostat experiments. Instead, we argue that NH_2_OH production has a role in the secondary metabolite metabolism of *A. faecalis*.

The *dnf* gene cluster encodes several proteins annotated as amino- and hydroxymethyltransferases that were all up-regulated in NH_4_^+^ excess conditions, suggestive of a biosynthetic pathway. This is in line with the results obtained by Xu and colleagues (*29*), who were unable to demonstrate that DnfA binds NH_4_^+^. It rather indicates that DnfA does not directly oxidize NH_4_^+^ (or NH_3_) but rather an amino group bound to an organic carrier molecule. Several classes of bioactive metabolites have been shown to contain NH_2_OH moieties. Hydroxamic acids, for example, are widely found as functional groups of bacterial antibiotics (*64*) and siderophores (*65*). *A. faecalis* has also been reported to produce hydroxamate-containing siderophores (*66*). However, the biosynthesis of siderophores seems unlikely in our experiments, considering that *A. faecalis* was cultured with an excess of iron. Furthermore, the production of hydroxamates does not explain the free NH_2_OH measured in the culture supernatant. Here, it is interesting to consider reports on a nitrifying *Arthrobacter* sp. that was found to secrete both free NH_2_OH and hydroxamic acid simultaneously (*67*). While NH_2_OH production was mainly correlated to the nitrogen concentration, hydroxamic acid was the main product in iron-deficient media. To the best of our knowledge, the effect of iron deficiency on NH_2_OH production in *A. faecalis* has not been assessed. On the other hand, recombinant *Escherichia coli* expressing DnfAB was capable of NH_2_OH production (*45*). DnfA contains a protein families database domain of the AurF family (PF11583), which was found to sequentially oxidize the amino group of aminobenzoic acid to nitrobenzoic acid, via a hydroxylamino intermediate (*68*). Moreover, the biosynthesis of trichostatin A in *Streptomyces* sp. RM72 involves an AurF homolog that converts the amido group of glutamine into hydroxylamine, yielding glutamic acid monohydroxamate (*69*). Thus, assuming a similar reaction mechanism in DnfA and a common precursor such as glutamine, it becomes apparent why recombinant *E. coli* is capable of NH_2_OH production with just DnfAB (*45*). These results point toward a precursor that is common to both *A. faecalis* and *E. coli* and is an amide-containing metabolite of a central metabolic pathway, such as certain amino acids. It has recently been reported that in vitro preparations of DnfAB are capable of hydroxylating the amide group of glutamine to form glutamic acid monohydroxamate, as is the case for TsnB7 (*48*). However, this still does not explain the release of hydroxamate moieties as free NH_2_OH.

The release of free NH_2_OH could be catalyzed by DnfC. Deletion of *dnfC* in *A. faecalis* JQ135 practically eliminated its ability to produce NH_2_OH (*29*). In addition, recombinant *E. coli* produced substantially more NH_2_OH with *dnfABC* than with *dnfAB* alone (*45*). The *dnfC* gene product is annotated as a putative glutamine amidotransferase and contains the type 1 GATase domain (PF00117). Amidotransferases catalyze the hydrolysis of an amido group to free and transfer NH_3_ to a receiving molecule. Under normal circumstances, this transfer is tightly regulated by binding of the receiving ligand, which prevents NH_3_ from leaking out (*70*). Nonetheless, the introduction of mutations can perturb this regulation and increase the leakage of free NH_3_ (*70*, *71*). Recently, *tsnB9* of *Streptomyces* sp. RM72 was found to be mistakenly annotated as an amidotransferase and rather function as hydroxyamidotransferase (*69*, *72*). Similar to amidotransferases, TsnB9 hydrolyzes the hydroxamate moiety of glutamic acid monohydroxamate to free NH_2_OH, transferring it to another carrier. Unfortunately, the authors did not report if free NH_2_OH was measurable. Nonetheless, it is probable that DnfC works in a similar fashion to TsnB9 and liberates NH_2_OH from an organic carrier. This activity has recently been reported for in vitro preparations of DnfC from *A. faecalis*, which were capable of hydrolyzing both the amide bond of glutamine and the hydroxamate group of glutamic acid monohydroxamate (Fig. 3) (*48*). To the best of our knowledge, this would be the second reported instance of a hydroxyamidotransferase and could have important implications for the phylogenetic diversity in which this activity is found.

The question remains why energy is invested in the production of NH_2_OH if there is no apparent growth benefit. Speculatively, but with several reports supporting this notion, the physiological benefit may be related to the cytotoxic effects of NH_2_OH. The inhibitory properties of NH_2_OH produced by heterotrophic nitrifiers were first observed in *Arthrobacter* sp. (*73*). Later, NH_2_OH production by *A. faecalis* was also shown to have antifungal (*74*, *75*) and antibacterial (*76*, *77*) effects. NH_2_OH secretion could, therefore, be a competitive strategy. *A. faecalis* grown in defined cocultures had a competitive advantage over its concomitant organisms, which the authors ascribed to the production of NH_2_OH (*77*). Furthermore, *A. faecalis* has been suggested as a possible plant growth-promoting bacterium. This has been the case in *Citrus sinensis*, where inoculation with *A. faecalis* reduced the virulence of *Xanthomonas citri* subsp. citri (*50*). In another case, cabbage seedlings inoculated with *A. faecalis* were less susceptible to clubroot, a disease caused by the fungus *Plasmodiophora brassicae* (*78*)*.*

In conclusion, we show that NH_2_OH is the main product of heterotrophic nitrification by *A. faecalis*. We demonstrated that N_2_O and N_2_ production are mainly explained by abiotic processes. No clear indications were found for either denitrification or direct NH_4_^+^ oxidation to N_2_, although small amounts of NO_2_^−^ reduction apparently contributed to N_2_O production. Given the body of research attempting to apply heterotrophic nitrification to wastewater treatment systems [reviewed in (*79*)], this lack of HNAD and biotic NH_2_OH oxidation under nitrifying conditions is annihilating this process idea. If our findings can be generalized to other heterotrophic nitrifiers, then the copious production of NH_2_OH and N_2_O would make it unsuitable for use in sustainable wastewater treatment. Therefore, careful examination of abiotic NH_2_OH conversion mechanisms should be considered when characterizing newly isolated heterotrophic nitrifiers or testing their applicability to wastewater treatment (*80*).

Together, our data indicate that heterotrophic nitrification in *A. faecalis* is in fact restricted to the production of NH_2_OH, with ensuing NO_2_^−^ production because of spontaneous formation and subsequent oxidation of pyruvic oxime and abiotic reactions that form N_2_O and N_2_. Further investigation is required to uncover the exact mechanism of NH_2_OH production and if an organic precursor is involved. Possibly, the production of free NH_2_OH could be a competitive strategy for heterotrophic bacteria.

## MATERIALS AND METHODS

### Cultivation and medium

*A. faecalis* ATCC 8750 was obtained from the ATCC and maintained in Difco Nutrient Broth (NB; BD Diagnostics) at 30°C and shaking at 200 rpm or on 1.5% agar plates at 37°C. Nitrification experiments were performed in MM containing per liter dH_2_O:0.5 g of NH_4_Cl, 0.2 g of KH_2_PO_4_, 0.04 g of MgSO_4_ × 7 H_2_O, and 0.2 mg of CaCl_2_ × 2 H_2_O. MM was amended with Fe-NTA solution (1 ml liter^−1^) and trace element solution (1 ml liter^−1^), as described by Sakoula *et al.* (*81*).

### Genome analysis

The genomic DNA of *A. faecalis* ATCC 8750 was isolated from NB-grown cultures using the DNeasy PowerSoil Kit (QIAGEN), according to the manufacturer’s instructions. Illumina short-read libraries were prepared and sequenced by Macrogen Europe using the TruSeq DNA PCR Free Kit (Illumina). The raw sequencing data were quality-checked using FastQC v0.11.5 (*82*) and trimmed using Flexbar v2.7 (*83*). Long-read libraries for Oxford Nanopore sequencing were prepared with the Ligation Sequencing Kit (SQK-LSK109, Oxford Nanopore Technologies) and sequenced with a FLO-MIN106 flow cell on the MinION device. Base calling was performed using Guppy v6.1.5 in flip-flop mode. Adapters were removed using Porechop v0.2.3 (https://github.com/rrwick/Porechop). Hybrid genome assembly was done by Unicycler v.0.4.4. (*84*) with default settings. Genome quality was checked using CheckM2 (*85*). Gene calling and annotation were done using a combination of the MicroScope platform (*86*), DRAM (*87*), and TransportDB v2.0 (*88*). Manual curation of the annotation for genes involved in the assimilation and metabolism of inorganic nitrogen was done by searching for homologous genes (≥35% shared identity) in the SwissProt database and comparing gene synteny with published gene clusters.

### Influence of C/N ratio in batch

*A. faecalis* was grown overnight in MM medium containing 20 mM Na_2_-succinate, 10 mM NH_4_Cl, and 50 mM Hepes (pH 7.2) at 30°C with shaking at 150 rpm. This preculture was used to inoculate (to an OD_600_ of 0.002) 120-ml serum vials containing 20 ml of MM containing 50 mM Hepes (pH 7.2), 10 mM Na_2_-succinate and 10 mM (C/N 4), 5 mM (C/N 8), or 2 mM (C/N 20) NH_4_Cl, with or without addition of 1 and 3 mM NH_2_OH-HCl. The serum vials were crimp-sealed with thin gray bromobutyl rubber stoppers. Automated high-resolution gas profiles were obtained as described previously (*89*, *90*) at 30°C and 700 rpm stirring.

### Origin and fate of NH_2_OH

*A. faecalis* was grown on NB and transferred to grow overnight on MM, centrifuged 30 min at 1500*g*, resuspended in sterile MM, and inoculated at an OD_600_ of 0.2. Incubations with ^15^N isotopes were performed in 120-ml serum vials containing 20 ml of MM, 5 mM Na_2_-succinate, and 25 mM Hepes (pH 7.2) and were crimp-sealed with thick red bromobutyl rubber stoppers. Incubations were supplemented with combinations of 2.5 mM NH_4_Cl, 0.5 mM NaNO_2_, and, after 1.5 hours, 0.25 mM NH_2_OH-HCl. ^15^N variants of these substrates were added as ^15^NH_4_Cl (99%), Na^15^NO_2_ (98%), and ^15^NH_2_OH-HCl (98%; Cambridge Isotope Labs) in the same concentrations. Control incubations were performed using replicates with sterile MM or by the addition of heat-sterilized biomass instead of active cells.

Abiotic reactions between NH_2_OH and NO were tested in duplicate in 20 ml of sterile MM or ddH_2_O amended with 5 mM Na_2_-succinate, 25 mM Hepes (pH 7.2), 2.5 mM ^14^NH_4_Cl, 0.06% (v/v) headspace NO, and with or without the addition of 0.25 mM ^15^NH_2_OH.

### Chemostat experiments

Cells were grown in a 1 liter of chemostat stirred at 800 rpm at pH 7.2, receiving 2.6 liter of MM day^−1^ and 0.16 liter of substrate solution day^−1^. The substrate solution consisted of 250 mM Na_2_-succinate and 250 mM (C/N 4), 125 mM (C/N 8), or 50 mM (C/N 20) NH_4_Cl. Oxygen supply was controlled at 20% O_2_ saturation. At each C/N ratio, triplicate samples were taken for RNA isolation; C, H, N, and O determination; protein determination; and colorimetric assays of NH_4_^+^, NH_2_OH, NO_2_^−^, and NO_3_^−^. Samples for RNA isolation were pelleted 5 min at 20,000*g* at 4°C. Immediately, the pellet was snap-frozen in liquid nitrogen and stored at −70°C.

### Analytical techniques

NO_3_^−^, NO_2_^−^, and NH_4_^+^ were measured photometrically according to Garcia-Robledo *et al.* (*91*) and Taylor *et al.* (*92*), respectively. NH_2_OH was measured according to Frear and Burell (*93*). Isotopes of N_2_, N_2_O, NO, CO_2_, and O_2_ were measured by gas chromatography and mass spectrometry using an Agilent 6890A/5975C and Agilent 8890A/5977B, both equipped with an Agilent 6 FT Porapak Q 80/10, by injecting 50 μl of gas directly from the headspace at each time point. CHNO content was determined using a vario MICRO cube (Elementar). The protein content was determined using the bicinchoninic acid Protein Assay Kit (Thermo Scientific Chemicals).

### Transcriptome analysis

RNA was isolated using the RiboPure-Bacteria Kit (Life Technologies Corporation). All centrifugation steps were performed according to the manufacturer’s instructions at 18,000*g* at 4°C, except for the final elution step and deoxyribonuclease treatment, which were performed at room temperature. Ribosomal RNA depletion and library construction were performed by Macrogen Europe using the Bacterial rRNA Depletion Kit (New England Biolabs) and TruSeq Stranded Total RNA Kit (Illumina), respectively. The raw Illumina data were treated as described above.

Trimmed reads were mapped against the assembled genome using Kallisto v0.46.2 (*94*). The alignment data from Kallisto were imported into R using tximport, and the differential gene expression analysis was performed using the DESeq2 workflow (*95*). Genes were considered differentially expressed between two conditions when their log_2_ fold change was ≥1.5 and FDR ≤ 0.05.
