# Supplementary material for: Hydroxylamine production by Alcaligenes faecalis challenges the paradigm of heterotrophic nitrification
Source: Sci Adv. 2024 Jun 7;10(23):eadl3587. doi: 10.1126/sciadv.adl3587 (PMC11160463; doi:10.1126/sciadv.adl3587)
Supplement: Supplementary file 1 — Figs. S1 to S4 Legend for table S1 [file sciadv.adl3587_sm.pdf]

Science Advances
AAAS

Supplementary Materials for

Hydroxylamine production by *Alcaligenes faecalis* challenges the paradigm of heterotrophic nitrification

Wouter B. Lenferink *et al.*

Corresponding author: Sebastian Lücker, s.luecker@science.ru.nl

Sci. Adv. **10**, eadl3587 (2024)
DOI: 10.1126/sciadv.adl3587

The PDF file includes:

Figs. S1 to S4
Legend for table S1

Other Supplementary Material for this manuscript includes the following:

Table S1

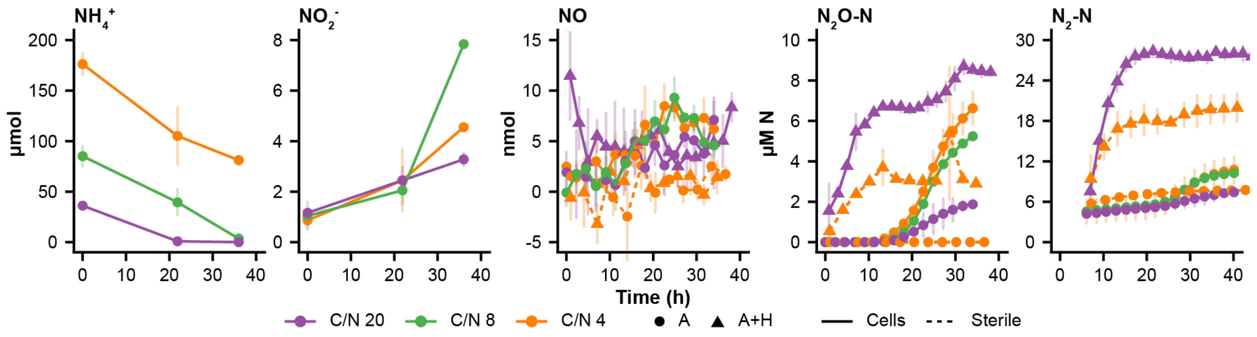

Fig. S1.

NH_4^+ consumption and subsequent production of NO_2^-, NO, N_2O, and N_2 by *A. faecalis*. Colors indicate the C/N ratio condition, closed circles represent incubations with NH_4^+ as the sole nitrogen source (condition A), closed triangles represent incubations with additionally 1 mM (orange) or 3 mM (purple) NH_2OH added (condition A+H). Solid lines represent incubations with *A. faecalis*, and dotted lines experiments in sterile medium. Data points and error bars represent the mean and standard deviations, respectively, of three biological replicates.

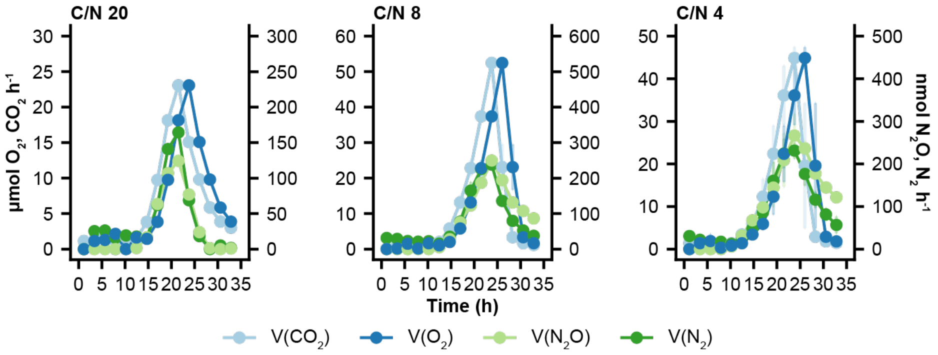

Fig. S2.

Rate kinetics of CO_2, N_2O, and N_2 production and O_2 consumption by *A. faecalis* in batch culture. Incubations were performed at starting C/N ratios 20 (left), 8 (middle), and 4 (right). Data points and error bars represent the mean and standard deviations, respectively, of three biological replicates.

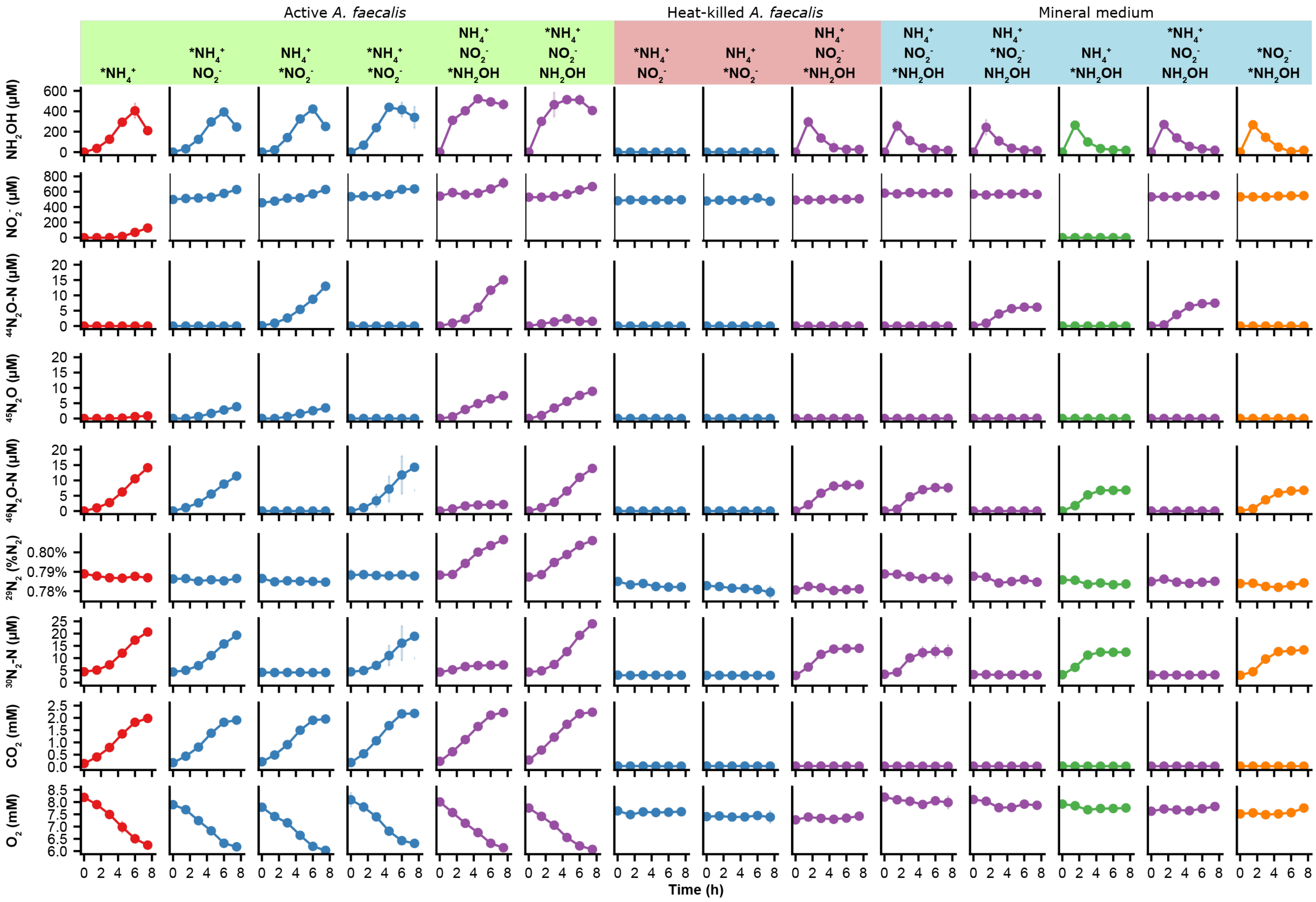

Active A. faecalis
Heat-killed A. faecalis
Mineral medium
*NH4+
*NH4+ NO2-
NH4+ *NO2-
*NH4+ *NO2-
NH4+ NO2- *NH2OH
*NH4+ NO2- NH2OH
*NH4+ NO2-
NH4+ *NO2-
NH4+ NO2- *NH2OH
NH4+ NO2- *NH2OH
NH4+ *NO2- NH2OH
NH4+ *NH2OH
*NH4+ NO2- NH2OH
*NO2- *NH2OH
NH2OH (μM)
NO2- (μM)
44N2O-N (μM)
45N2O (μM)
46N2O-N (μM)
29N2 (%N2)
30N2-N (μM)
CO2 (mM)
O2 (mM)
Time (h)

Fig. S3.

Measurements of CO_2, O_2, and nitrogen compounds in batch incubations with *A. faecalis*, heat-killed *A. faecalis*, or mineral medium. Line colors indicate which nitrogen compounds were added (column headers, vertical). Asterisks in the column headers indicate the nitrogen compounds added as ^{15}N stable isotope. Data points and error bars represent the mean and standard deviations, respectively, of three biological replicates.

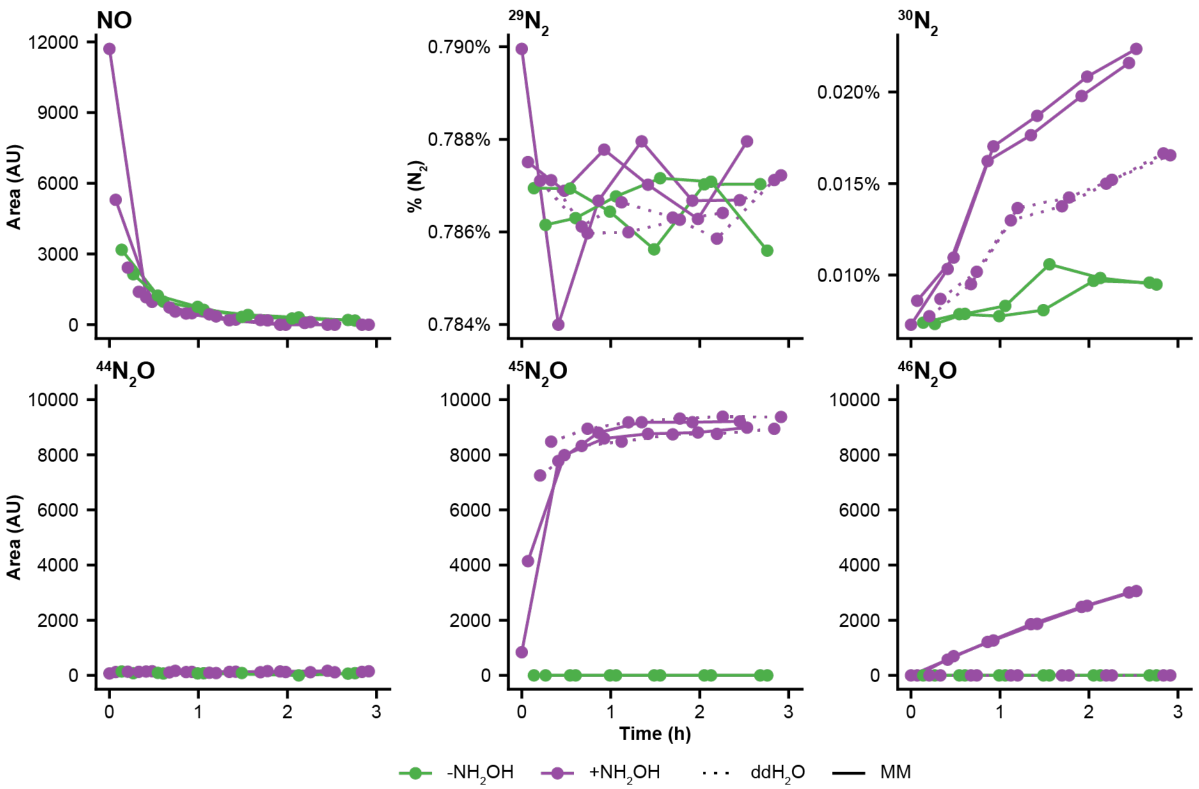

Fig. S4.

Abiotic reactions of NO with and without NH_2OH to N_2O and N_2. Shown are incubations with 0.06% (v/v) NO only (green lines) and with 0.06% (v/v) NO and 0.25 mM ^{15}N-labelled NH_2OH (purple lines), either in demineralized water (dotted lines) or in mineral medium (solid lines). Note that $^{28}N_2$ formation was not determined. Duplicate experiments are shown as separate curves.

Table S1. (separate file)

Log2-fold changes (LFC) of genes that showed significant differential gene expression (LFC > 1.5, FDR < 0.05) in any of the pairwise comparisons between conditions C/N 20, 8, and 4. Frame, begin, and end columns are relative to AFN_v2_0001. Gene and product columns are a combination of manual and automatic annotations using the MicroScope annotation platform (see methods).
